# Complete hazard ranking to analyze right-censored data: An ALS survival study

**DOI:** 10.1371/journal.pcbi.1005887

**Published:** 2017-12-18

**Authors:** Zhengnan Huang, Hongjiu Zhang, Jonathan Boss, Stephen A. Goutman, Bhramar Mukherjee, Ivo D. Dinov, Yuanfang Guan, for the Pooled Resource Open-Access ALS Clinical Trials Consortium

**Affiliations:** 1 Department of Computational Medicine and Bioinformatics, University of Michigan, Ann Arbor, MI, United States of America; 2 Department of Biostatistics, University of Michigan, Ann Arbor, MI, United States of America; 3 Department of Neurology, University of Michigan, Ann Arbor, MI, United States of America; 4 Department of Health Behavior and Biological Sciences, University of Michigan, Ann Arbor, MI, United States of America; 5 Statistics Online Computational Resource, University of Michigan, Ann Arbor, MI, United States of America; 6 Michigan Institute for Data Science, University of Michigan, Ann Arbor, MI, United States of America; 7 Department of Internal Medicine, University of Michigan, Ann Arbor, MI, United States of America; 8 Department of Electronic Engineering and Computer Science, University of Michigan, Ann Arbor, MI, United States of America; Philadelphia, UNITED STATES

## Abstract

Survival analysis represents an important outcome measure in clinical research and clinical trials; further, survival ranking may offer additional advantages in clinical trials. In this study, we developed GuanRank, a non-parametric ranking-based technique to transform patients' survival data into a linear space of hazard ranks. The transformation enables the utilization of machine learning base-learners including Gaussian process regression, Lasso, and random forest on survival data. The method was submitted to the DREAM Amyotrophic Lateral Sclerosis (ALS) Stratification Challenge. Ranked first place, the model gave more accurate ranking predictions on the PRO-ACT ALS dataset in comparison to Cox proportional hazard model. By utilizing right-censored data in its training process, the method demonstrated its state-of-the-art predictive power in ALS survival ranking. Its feature selection identified multiple important factors, some of which conflicts with previous studies.

## Author summary

We present a novel rank-based algorithm that outputs the survival likelihood of the ALS patients from the survival data. This novel method enabled us to adopt commonplace machine learning base-learners in survival analysis of ALS and provided insight into the disease.

## Introduction

Survival analysis is essential in clinical research. To assess efficacy and safety of a novel therapy on human subjects, researchers need to carry out clinical trials and collect longitudinal data from participants [1]. To reliably and robustly estimate the effect of a new therapy scheme on subjects' survival, survival analysis is required. From massive time-event data from clinical trials, survival analysis identifies factors that are predictive to subjects' lifetime and estimates survival time for new subjects. Similar information cannot be retrieved from simple mortality comparison, which makes survival analysis necessary and irreplaceable [2].

The most widely used survival prediction model nowadays is Cox proportional hazards (PH) model [3]. The model assumes that the hazard ratio is a function over a linear combination of multiple factors and that factors of interest are independent of time. Validating the assumptions is important and inappropriate adoption of the Cox model might lead to erroneous predictions [4–6]. Since then, researchers have proposed variations of the Cox model with relaxed assumptions [7–11] and alternative statistical models such as the Accelerated Failure Time model [12–15]. The original Cox model and its variants have been adopted in all areas to this day, and researchers continue to improve them for different scenarios.

Recent advances in machine learning have brought alternative solutions to survival analysis. Machine learning regression algorithms rarely require extensive manual parameter tuning [16], yet they cannot handle censored subjects, events of which were not observed. Because of this, researchers proposed to use Kaplan-Meier estimator [17] to infer probabilistic survivability for censored cases and transform the problem into a general regression problem [18–21]. Recently, random survival forest demonstrated better performance than many previous machine learning models and showed promise in survival analysis [22]. The algorithm builds an ensemble of binary survival trees [23], which is, unfortunately, specific to tree models and cannot be easily applied to other regression methods. A strategy that allows regression algorithms to generate fine-grained survival predictions would be useful to the field.

To leverage the power of commonplace machine learning algorithms in survival analysis, we developed a novel hazard ranking algorithm named “GuanRank” and a rank-based survival model. The ranking algorithm ranks all training subjects based on their relative hazards with help of the Kaplan-Meier function [17]. Machine learning algorithms then build regression models between the factors and ranks. To evaluate the method, a proof-of-concept model was built on the Pooled Resource Open-Access Amyotrophic Lateral Sclerosis (ALS) Clinical Trials (PRO-ACT) database, a large de-identified dataset of ALS clinical trials [24]. The model was benchmarked using cross-validation tests and independently evaluated in DREAM ALS Stratification Prize4Life Challenge. Ranked first place, the model predicted survival of individual patients with a survival concordance index of 0.717. Several important factors were identified by the model, some of which conflicts with previous studies.

## Results

### The hazard ranking algorithm and the survival prediction method

**Fig 1:**
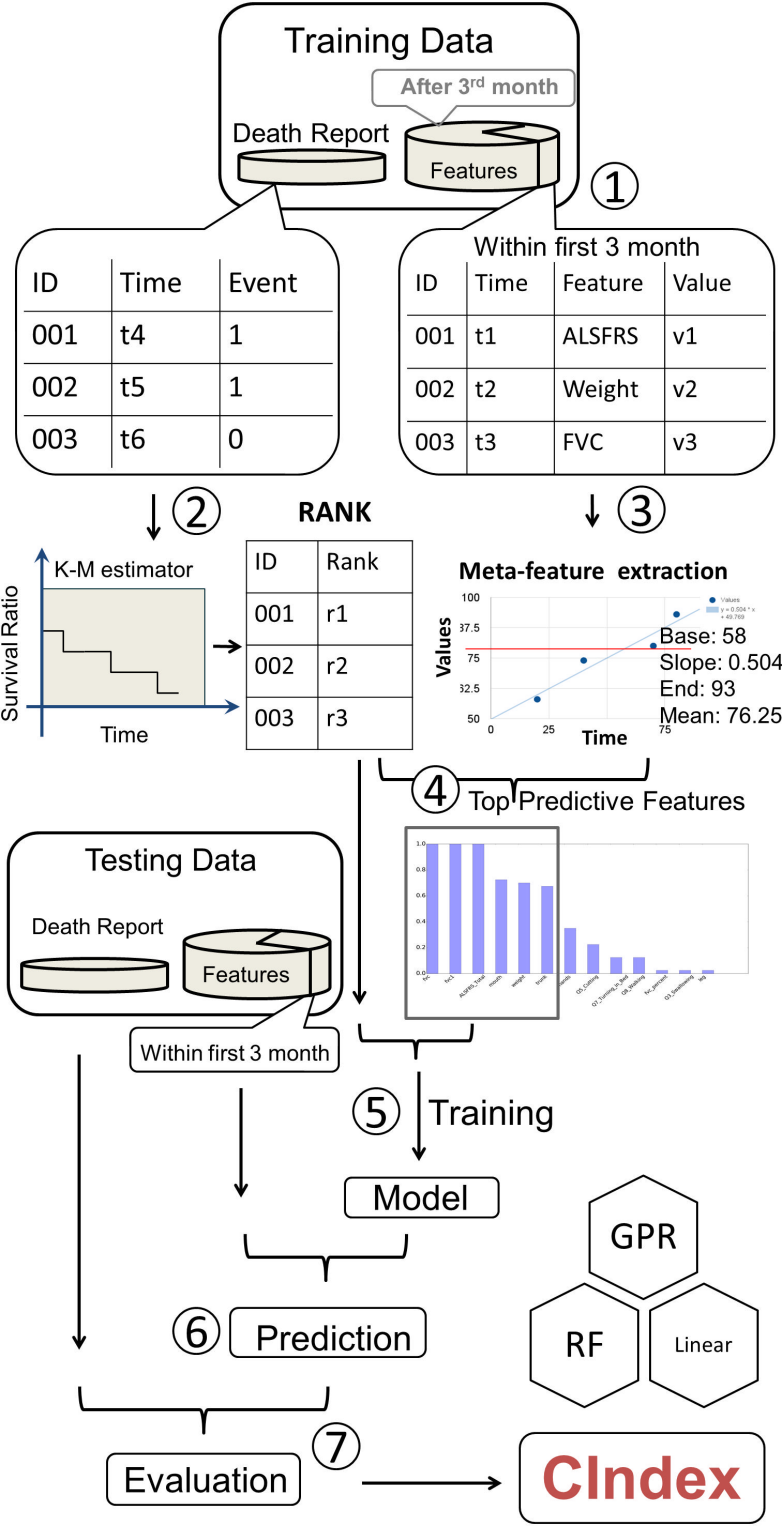
Workflow. Data were randomly divided into training and testing datasets for both survival status and clinical features. Because the challenge evaluation used first 3-month data as the prediction input data, only the features recorded during the first 3 months of patient enrollment in the trial were included in the data training features. (Step 1) Using the survival data, and K-M estimator, we ranked all the individuals and assigned each one ranking score. (Step 2) Each feature was further extracted to four meta-features. (Step 3) After calculating the correlation between the meta-features and survival results, we chose the top related features to be counted as the training and testing data. (Step 4) The training ranking was set as the training target to generate the model. This model was subsequently applied to the testing features for prediction. The prediction results were compared to test death report data for concordance Index shown as ‘C’ in the figure (right bottom).

To bring the power of common machine learning regression methods to survival analysis, we proposed our workflow shown in Fig 1. The algorithm extracts risk factors (or features) from the dataset and generates meta-features to describe the trends or the characteristics of raw features, such as means, minima, maxima, or linear regression slopes. A Kaplan-Meier survival function is then estimated on the whole dataset. Based on that, the algorithm assigns a hazard rank for each subject and selects factors that are highly correlated with the ranks to build prediction models. Common regression algorithms are fitted to the hazard ranks using selected features, and the best prediction model is determined through cross-validation tests.

One major challenge to incorporate machine learning algorithms in survival prediction is to handle censored subjects, survival time of which are unknown. Because regression algorithms require complete prediction targets for all subjects, regression algorithms cannot be directly fitted to the survival time. The proposed workflow includes a complete hazard ranking algorithm to address this problem: the ranking algorithm assigns hazard ranks to all subjects (including censored ones), and a machine learning model can then be fitted to the hazard ranks. To calculate hazard ranks, the ranking algorithm performs a complete hazard comparison for all pairs of subjects in the training set. Subjects with clear earlier event occurrence are assigned higher ranks. For censored subjects, the algorithm uses Kaplan-Meier survival function to estimate their hazard levels and assigns ranks accordingly. Every subject receives a hazard rank after the complete comparison.

Once the complete hazard ranking is done, the machine learning models can be then fitted to the complete ranks instead of the incomplete survival time. Various machine learning regression algorithms, including Lasso regression, Gaussian process regression, and random forest regression, have been tested. The performance was evaluated in terms of the concordance index (c-index), the ratio of subject pairs with correctly predicted event occurrence order. A 20-time 2-fold cross-validation (20x2CV) test was performed to estimate the average performance of individual models, which was reported to be more accurate in estimating prediction performance [25].

### Development of PRO-ACT survival prediction model

To evaluate the rank-based survival regression method, a proof-of-concept ALS survival model was developed on a subset of the PRO-ACT dataset, a de-identified database including clinical variables and survival data for now over 10,000 ALS patients who participated in Phase 2 and 3 trials between 1990–2013 [24]. The subset was collected by DREAM Challenges organizers, and its patient-level demographic characteristics are summarized in Table 1. It contained de-identified medical records from 8,635 participants, 6,565 of which have complete demographic data, including gender, age, and race. The mean age was 56.22 years (standard deviation: 11.78 years). The gender ratio (male: female) was 1.58:1. Among patients with full demographic data, 4,803 (97.21%) subjects were listed as Caucasians. 77.53% of participants were riluzole users. The average time between the onset of symptoms and the trial entry was 709.66 days (± 539.27 days), while the average time between diagnosis and trial entry was 320.19 days (± 356.75 days). Baseline forced vital capacity (fvc) was 78.24% (± 24.05%).

**Table 1:** The summary of the PRO-ACT dataset used in this study.

Variable	Group/Unit	n	Mean(±SD) or %
Male:Female Ratio		6,565	1.58:1
Age	Years	6,565	56.22 ±11.78
Race	Caucasian	4,803	73.16%
	Unknown	1,624	24.74%
	America Indian	2	0.03%
	Asian	38	0.58%
	Black	68	1.04%
	Hispanic	18	0.27%
	Other	12	0.19%
3-month fvc percent	%	6,186	79.24 ±22.61
Riluzole users		5,511/7,108	77.53%
3-month BMI	kg/m^2^ (kg/cm^2^ *)	3,207	25.37 ±4.425
overall BMI	kg/m^2^ (kg/cm^2^ *)	3,207	25.37 ±4.416
3-month ALSFRS		4,835	29.03 ±5.89
Overall ALSFRS		4,838	26.21 ±6.38
Baseline ALSFRS	No ALSFRS_R_Total score available	3,102	29.46 ± 5.96
Baseline ALSFRS-R	With both ALSFRS_Total and ALSFRS_R_Total scores available	1,736	38.27 ± 5.29
Time between diagnosis and trial entry (Diag_delta)	Days	2,769	320.19 ±356.75
Time between symptom onset and trial entry (Onset_delta)	Days	4,985	709.66 ±539.27

The ‘unknown’ data was excluded from all the calculation. The 3-month data was used for the training of our model. BMI: Body Mass Index, fvc: Forced Vital Capacity, ALSFRS: ALS Functional Rating Scale, ALSFRS-R: ALS Functional Rating Scale Revised. Note that the BMI in this dataset is calculated by using cm and kg as units for height and weight, while the conventional calculation of BMI uses m and kg as units.

*: challenge data presented in these units

The complete hazard ranking algorithm and regression methods were then applied to the PRO-ACT dataset to build proof-of-concept ALS survival prediction models. As specified by the competition rules, only 6 features were allowed in the model. In this study, the feature with the highest correlation between a feature and the survival ranks in the training dataset were selected. Then, the following selection steps were iteratively performed until the maximum number of features was reached:

- Remove any remaining features that have correlation coefficients higher than 0.9 against any selected features;
- Select the feature, out of the remaining ones, with the highest (Pearson) correlation with the survival ranks.

**Fig 2:**
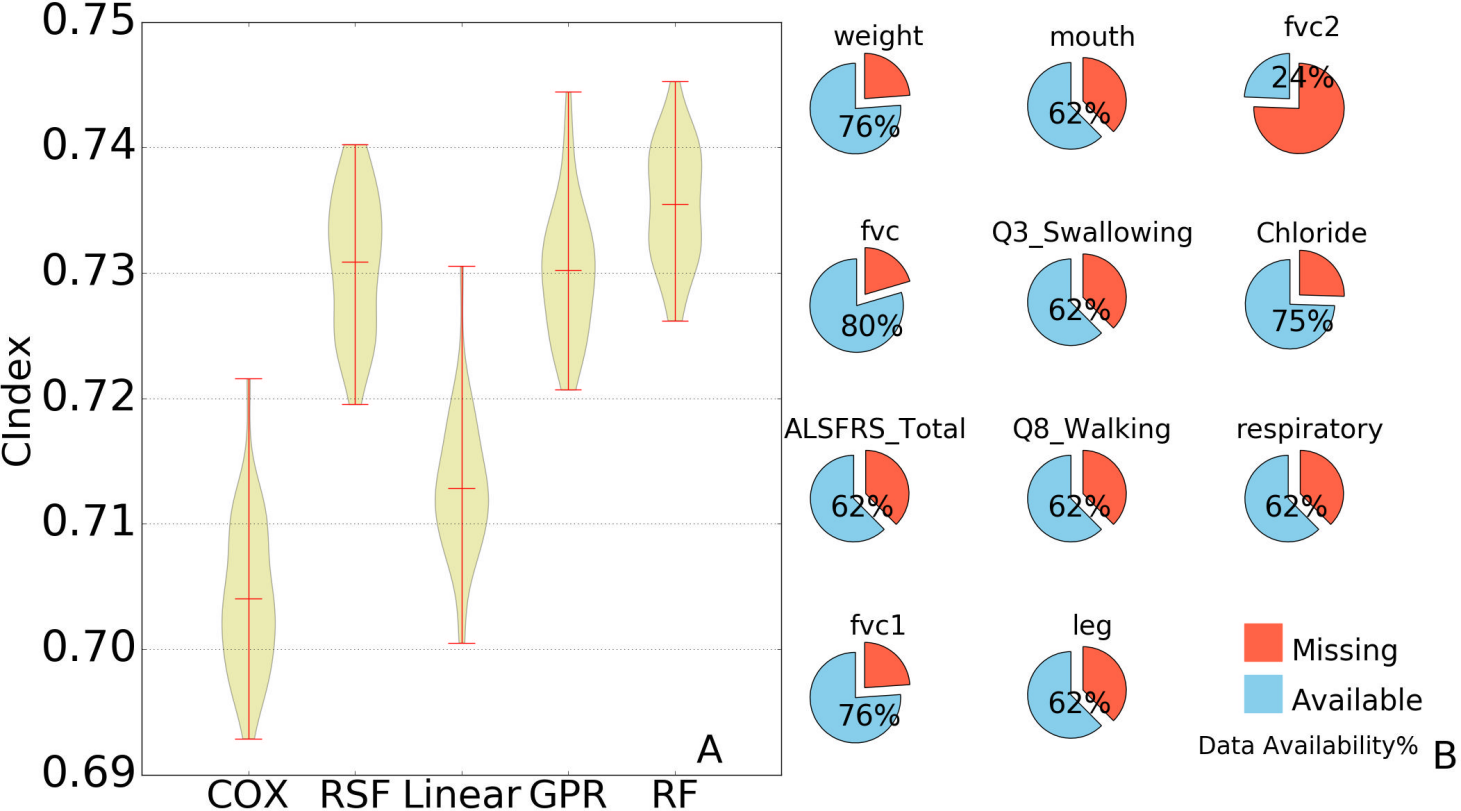
Comparison of performance and predictive features availability. A: The results of 2-fold cross validations are shown for each regression method in this in this figure, Cox: Cox proportional hazards regression model, RSF: random survival forest, GPR: Gaussian process regression, Linear: linear (Lasso) regression, and RF: random forest. For Cox model and random survival forest, the time-event data was used as the training target while our ranking values were used for the other 3 regression method. The distribution of concordance index is represented by the width of the violin shape: the wider the shape, the greater the sample concentrated. The three red horizontal lines in each violin shape shows the lowest, mean, and the highest concordance for each method. B: The eleven features used and their availability. Weight = weight in kg; mouth = composite score of ALSFRS questions 1–3; fvc1/fvc2 = value (in liter) of first/second attempt in fvc measurement, fvc = average of fvc values in measurement attempts; Q3_swallowing = ALSFRS Question 3 score; chloride = chloride concentration in blood; ALSFRS_total = sum of ALSFRS question scores; Q8_walking = question 8 of ALSFRS; respiratory = question 10 in ALSFRS and question 1 in ALSFRS-R; leg = composite scores combining ALSFRS question 8 to 9.

Selected features were then used to build prediction models. LASSO regression, random forest regression, and Gaussian process regression were tested. For the sake of comparison, survival models such as the Cox model and random survival forest were included in our tests. The performance was evaluated in c-index. A random and perfect prediction model would achieve c-indices of 0.5 and 1.0 respectively. The results are shown in Fig 2. Gaussian process regression achieved an average c-index of 0.7302, linear (LASSO) regression 0.7128, and random forest regression 0.7355. The Cox model yielded an average c-index of 0.7041, and random survival forest 0.7308. Out of all 40 rounds of tests, the rank-based random forest model outperformed the Cox model all 40 times.

The models were also evaluated in terms of their prediction accuracy of actual survival time based on cross-validation prediction errors. We proposed two survival time prediction methods based on the random forest rank-based: One way was to map the rank back to the Kaplan-Meier curve and to project their survival time directly from the Kaplan-Meier curve, and the other way was to linearly interpolate survival time from the hazard rank in the training dataset. The projection method would report the same survival time for subjects with ranks that lie between uncensored training samples, while the interpolation method would report different survival time from such ties. They were compared against the Cox model. A common practice of survival time prediction using the Cox model is to calculate median survival time. However, subjects with low risks may not have median survival time, and the prediction accuracy would be undermined. For the sake of fairness, a projection method was proposed for the Cox model similarly. Estimated hazard ratios of training and testing subjects were normalized to the range from 0 to 1. The survival time was estimated by mapping normalized hazard ratios to the inverse function of the training data Kaplan-Meier survival function. For all three methods, subjects with extremely low (predicted) risks were predicted to have the same survival time as the longest survival time observed in the training dataset. The performance was evaluated in terms of average absolute prediction error, restricted to only uncensored cases in the testing dataset. The Cox projection method achieved an average absolute error of 158 ± 3 days, the rank-based projection method 152 ± 4 days, and the interpolation method 140 ± 5 days. Out of 40 rounds of cross-validation tests, the rank-based projection method outperformed the Cox projection method 39 times, and the interpolation method outperformed the Cox projection method 40 times.

Based on the above tests, the best performer, the rank-based random forest regression model, was chosen as the final survival prediction model. The performance of the model was independently validated in the DREAM Challenge on an independent hidden dataset and achieved a c-index of 0.717.

### Inspection of the ALS survival prediction model

The ranking approach not only generated accurate prediction models, but its automatic feature selections can help identify key factors in the ALS survival as well. The above rank-based random forest model was inspected to identify highly ranked features chosen in the survival prediction. In each round of the 20x2CV tests, the program selected features independently. For convenience's sake, ALSFRS questionnaire features were listed in S1 Text. Throughout all 40 rounds of repeated cross-validation tests, 11 features were selected independently at least once (Fig 2B), including ALSFRS_Total (ALS Functional Rating Scale total score), fvc, weight, fvc1, mouth, Q3_swallowing, Q8_walking, leg, fvc2, chloride, and respiratory. Among these features, 3 were selected in every round of the tests (fvc, weight, and ALSFRS_total), which shows a strong and robust correlation against the patients' survival. Previous studies reported weight loss to be a poor predictor to ALS development [26,27]; yet it correlated well with the hazard ranks and was considered highly predictive in the rank-based model. ALSFRS_total, the sum of 12 measurements related to a patient’s physical mobility, was shown to be a critical predictive feature for the survival expectation in the study.

**Fig 3:**
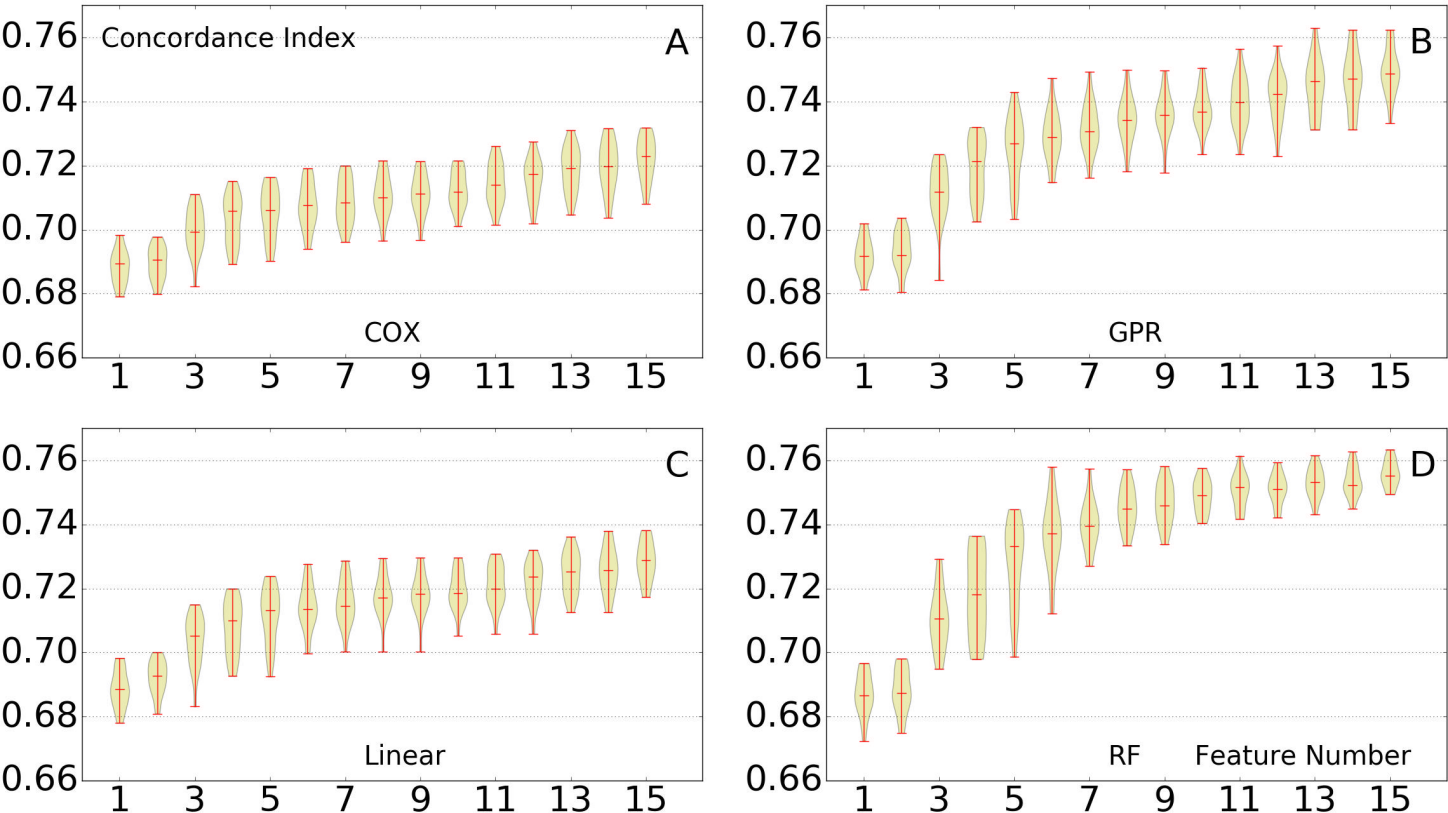
Testing of different feature numbers. The performance of all tested methods, as number of features is increased (x axis). Twenty rounds of 2-fold cross validation are performed using the 1 to 15 features. The performance gradually increases when we add features to the training set. The prediction performance significantly improved when the number of features adopted increases from 1 to 6. No closely related feature was excluded. (A) Cox model. (B) Rank-based Gaussian process regression. (C) Rank-based linear regression. (D) Rank-based random forest.

In the post-challenge analysis, the restriction on the number of features was no longer enforced. To examine the importance of more features, the models were to be tested with a larger number of features. But before that, the models were first tested with different number of features, to see whether their prediction performance was affected by changes of features. The rank-based survival models (with different regression algorithms) were tested with different number of features, ranging from 1 to 15. Similarly, the Cox model was included and tested similarly as a comparison. The results are shown in Fig 3. With the number of features increased, the performance of all prediction models improved accordingly. Yet the improvement slowed down once there were more than 6 features. Specifically, for the random forest regression model, the model with 15 features only marginally outperformed that with 10 features.

**Fig 4:**
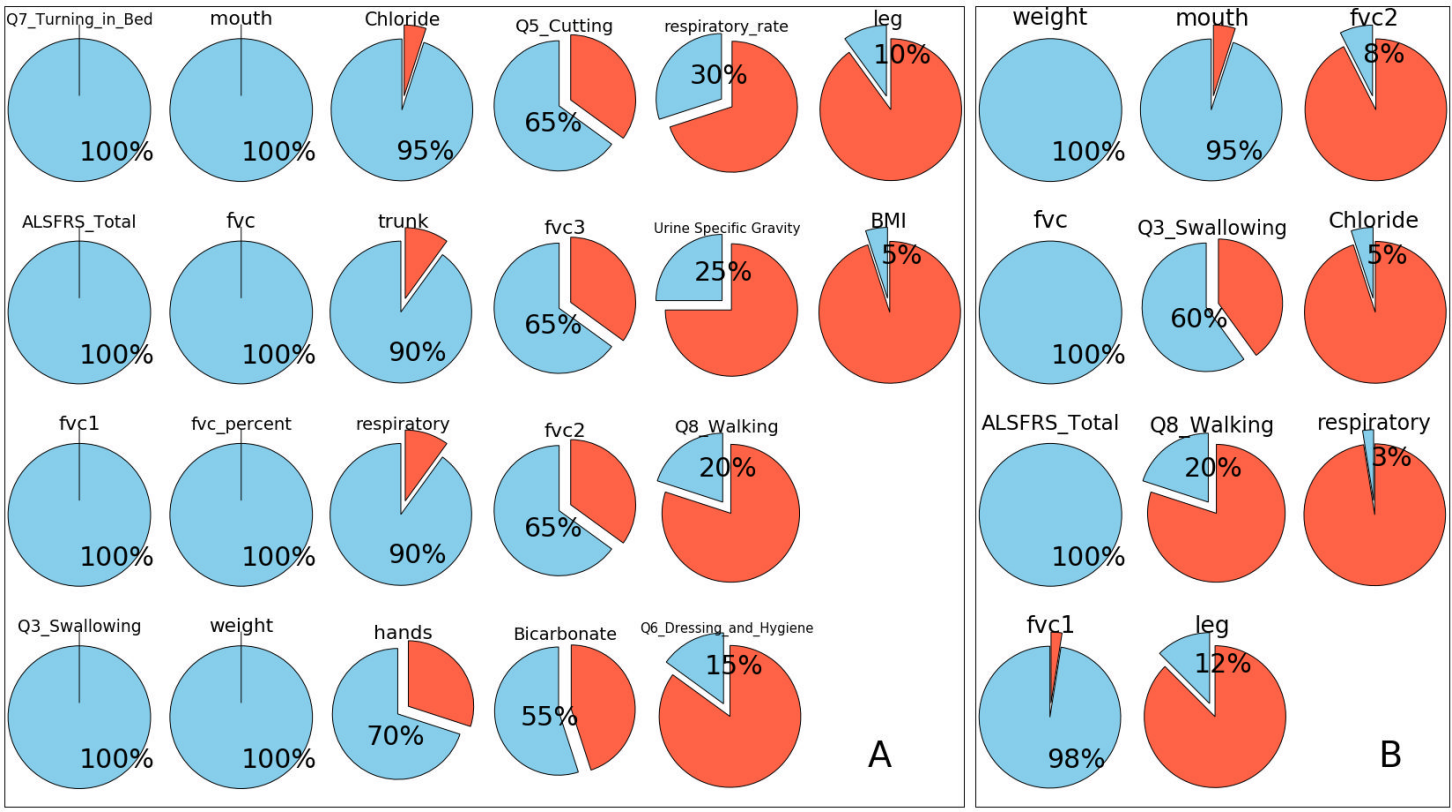
Feature adoption ratios in repeated cross-validation tests. The adoption ratio of a feature is, when the models are allowed to take only a limited number of features, the ratio of models that selects the feature throughout all 20 rounds of cross-validation tests. Since the data were split randomly throughout all the tests, the models might pick up a different set of features. The most significant features were expected to have high adoption ratios. Features such as fvc, ALSFRS Total, and weight were adopted in all training process; features like fvc1, mouth and Q3_Swallowing were picked in more than half of the tests. (A) Feature adoption ratios when models were allowed to take 15 features at most. In total 22 features have been picked at least once. (B) Feature adoption ratios when models were allowed to take 6 features at most. In total 11 features have been taken at least once.

After showing that the performance of the model was robust against changes of features, the model was then used to evaluate the importance of more features. In this section, a new rank-based random forest model was trained with 15 features and 20x2CV tests were performed. There were 22 features that were independently selected at least once throughout all 40 rounds of (repeated) cross-validation (Fig 4). Again, features such as fvc, weight and ALSFRS_Total were selected in all 40 rounds. Additionally, features such as Q7_turning_in_bed, fvc1, Q3 swallowing, mouth (Question 1 to 3), fvc percent (predicted) and weight, were included in all rounds of cross validations.

**Fig 5:**
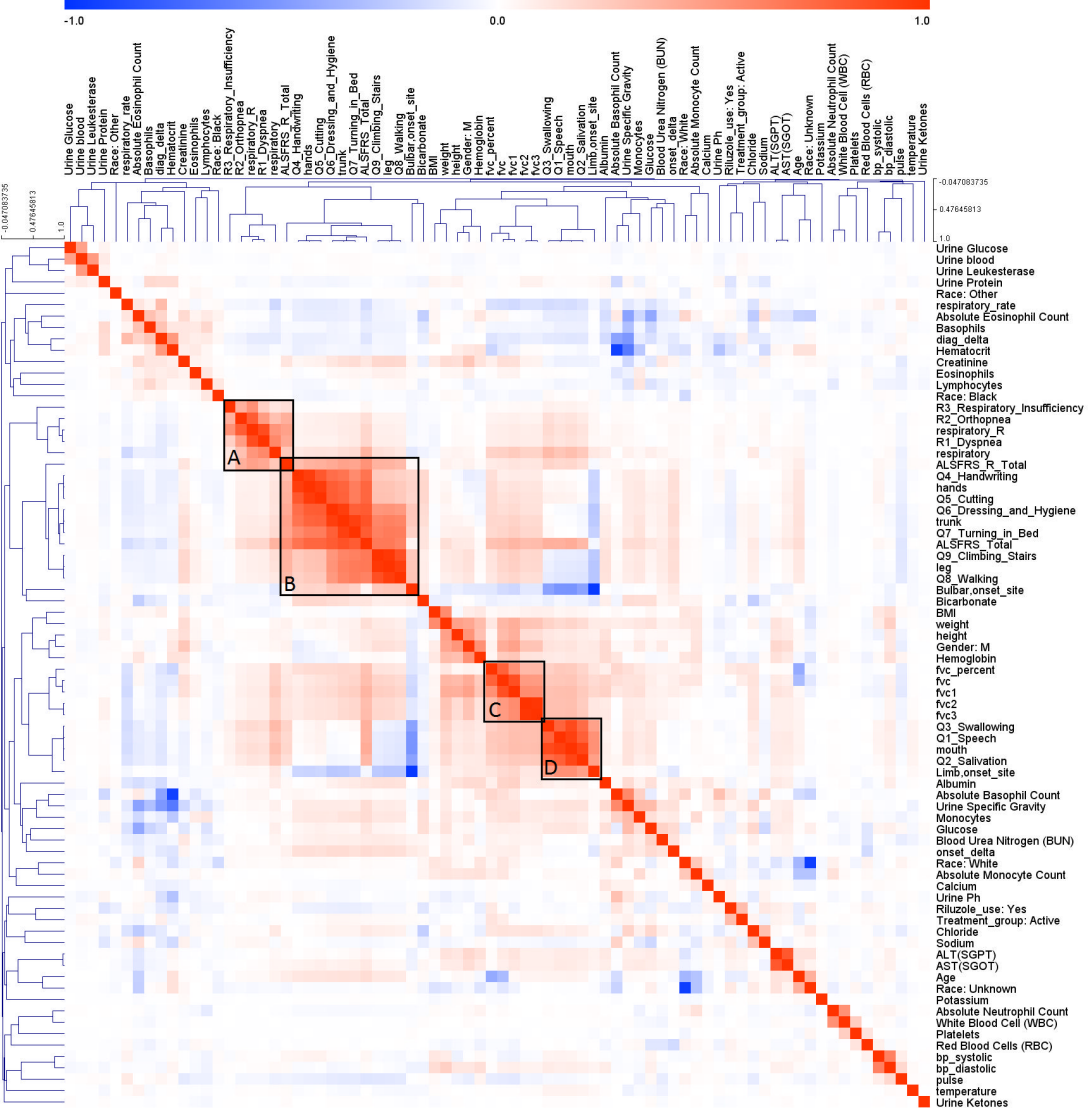
Features correlation. The colors in the heat-map show the Pearson correlation between each pair features. Dark color indicates a strong correlation, either positive (red) or negative (blue). Closely correlated features are clustered into group by applying hierarchical clustering. The black box shows several significant clusters among the features. A: Respiratory related features, B: Upper and lower limb related features and bulbar onset, C: FVC related features, D: Mouth related features and Limb onset.

As mentioned above, while the performance gradually increased with more features, the improvement was not significant. To see whether there were high correlations among top features, the correlation coefficients among features were plotted (Fig 5). Red and blue colors indicate strong positive and negative correlation between two features, respectively. Hierarchical clustering revealed closely related features. For example, part of the ALSFRS related features, shown in Box B, were positively correlated with bulbar onset. Another group of the ALSFRS related features, Q3_Swallowing, Q1_Speech, mouth and salivation, were clustered with limb (spinal) onset (Box D). This echoes previous reports on the distinctive characteristic of patients with limb and bulbar onsets [28].

## Discussion

Recent advances in survival analysis benefited from efforts in extending the Cox model and incorporating machine learning algorithms in survival modeling. Enlighted by previous efforts, we presented a novel non-parametric approach for survival ranking. Our method assigns hazard ranks to all subjects in the dataset and enables machine learning regression algorithms to build models between the observed factors of subjects and the hazard ranks. To validate our approach, we built a survival model on the PRO-ACT dataset, which is from a cross-sectional ALS longitudinal study. The method outperformed conventional models in our cross-validation tests, and the robust predictive power of our model was separately validated by DREAM Challenge on a hidden independent dataset from a different cohort. We inspected our ALS survival model and identified several highly predictive factors to ALS survival. We also compared our model with conventional survival models. Under different assumptions, they focus on different aspects of survival analysis.

### ALS survival analysis

By inspecting our ALS survival model, we summarized a few groups of predictive features to ALS survival. The ALSFRS and ALSFRS-R total score and the fvc are two features groups that strongly associate with ALS survival. It is expected that the ALS Functional Rating Scale, either the original or revised, is strongly associated with survival given that it is validated scale of physical function in ALS [29–31]. Previous studies have shown that trajectories of ALSFRS can be predictive to ALS survival [32]. Yet it is somewhat surprising that fvc (FVC in liters) was found more predictive than fvc_percent (FVC percent predicted). The latter is a more commonly used measure for trials, but the prediction data suggested that the unadjusted FVC was more predictive to ALS survival. This could suggest that baseline lung capacity is a prognostic feature. Q3_swallowing, which rates eating ability from 0 (exclusively parenteral or enteral feeding) to 4 (normal), and the feature summarizing the measure from ALSFRS questions 1 to 3 related to mouth functions were both considered as predictive features resulting from our cross validation. Q8_walking, which represents walking ability and meaningful leg movement, and the feature under the leg term, generated from the measurement of question 8 and 9 of the ALSFRS (or the revised ALSFRS), were shown less predictive. They, however, remained in the top predictive feature list. Question 10 in ALSFRS, breathing or Dyspnea in Revised ALSFRS, were merged under a feature named ‘respiratory’. Together with Age and Chloride, they also played a role in the survival prediction. This information may suggest that optimizing respiratory treatments can improve ALS disease management.

We noticed that the prediction model repeatedly selected weight, a feature not directly related to fvc and ALSFRS, to be a highly predictive factor in our cross-validation tests, contradicting with prior reports [26,27]. Also, although age was not even found predictive of survival in our tests on the PRO-ACT dataset, a younger age at disease onset is generally considered as a positive prognostic indicator [33,34]. The inconsistency might result from the heterogeneity of the affected population. For example, the average ages of ALS onset differ dramatically among studies carried out in different geographic regions or ethnic groups [35]. This could confound the impact of age for ALS survival analysis and potentially limit its application during survival prediction. Also, uric acid was considered as a predictive feature based on previous research [36], yet it was not selected in our study due to its low data completeness (35.51%).

Our study found that riluzole usage was an insignificant predictor of survival. It was also found by all top three DREAM challenge teams (https://www.synapse.org/#!Synapse:syn2873386/wiki/391432). Riluzole was reported to prolong survival by 2–3 months [37]. 77.53% of the samples in the training dataset are classified as riluzole users, according to the binary record, either ‘yes’ or ‘no’. This means that even if the patients used the drug for a limited time or inconsistently, they are classified as riluzole users. It is highly possible that preponderant short-term usage did not change the progression of the disease. Also, the PRO-ACT database may have included healthier ALS patients than the true ALS population [38]. This leads to the explanation that healthier patients are less dependent on the drug they used, therefore, riluzole does not play an important role in the survival prediction here.

### Survival ranking modeling

The core idea of our model is the complete hazard ranking algorithm. The idea of using a scalar value to summarize event occurrence was shown in previous studies [39,40]. Also, previous studies utilized Kaplan-Meier estimator for estimating survival for censored subjects [17]. In comparison, our method used a probabilistic scoring scheme for right-censored subjects, which were considered non-informative under the assumption of many previous models. Leaving out right-censored subjects in the analysis might lead to potential information loss in regard to survival rank predictions. The difference in dealing with right-censored subjects may have contributed to the high performance of our prediction model in this specific question.

Another important part of our model is the regression algorithm. Machine learning regression algorithms, such as random forest regression, do not require extensive parameter tuning and thus make the modeling process much easier. At the same time, methods like random forest or Gaussian process are able to handle nonlinearity in the data, making it possible to model the interaction between factors without explicitly specifying underlying distributions of the factors. This capability of nonlinear modeling is unseen in conventional linear models [41,42]. The regression algorithms would allow more flexible modeling, especially for cases that prior knowledge about event occurrence is limited.

By adopting machine learning regression algorithms, our model took a different approach to address the survival prediction. Instead of using the survival function output, the probability of surviving beyond certain time point directly, we use the newly developed rank output as the target of regression. This allows the transformation of the initial survival problem into probabilistic framework amenable to a variety of model-based and model-free predictive analytics [43,44]. In a different representation, our model compares the survivability of these subjects to known subjects in the database.

### Limitations

Despite the interesting results of this study, potential limitations are worth noting. One limitation of our study is that our method has not been tested on datasets other than those provided in the challenge. Even specifically for ALS survival analysis, the PRO-ACT dataset may not represent the true ALS population. Instead, it may include healthier and more homogenous participants [38]. Although our algorithm has been independently tested on a different cohort in the challenge, more tests are still needed to demonstrate the robustness of our approach. This does not only apply to ALS survival analysis, but also other diseases. A more comprehensive evaluation on multi-sectional datasets that truthfully demonstrate the heterogeneity of a disease may further help to show the potentials and benefits of our method, and applications of our methods on different diseases can be interesting. Further validation with prospective clinical datasets is warranted.

### Conclusions

In sum, we proposed a novel approach to rank subjects' hazard, which allows us to transform survival analysis problem to machine learning regression problem. The approach was separately tested by us and DREAM Challenge on two independent clinical datasets and achieved better performance than conventional algorithms. The complete hazard ranking algorithm allowed us to enroll right-censored subjects in our survival modeling, and the machine learning algorithms gave us a flexible way of nonlinear modeling over factors without explicitly specifying underlying distributions. The innovative approach showed promise in ALS survival analysis. More comprehensive studies of our approach on different diseases or cohorts would be potentially beneficial.

## Materials and methods

### Preprocessing of PRO-ACT dataset

We utilized the PRO-ACT dataset (https://nctu.partners.org/ProACT). Data used in the preparation of this article were obtained from the Pooled Resource Open-Access ALS Clinical Trials (PRO-ACT) Database. In 2011, Prize4Life, in collaboration with the Northeast ALS Consortium, and with funding from the ALS Therapy Alliance, formed the Pooled Resource Open-Access ALS Clinical Trials (PRO-ACT) Consortium. The data available in the PRO-ACT Database have been volunteered by PRO-ACT Consortium members. This research was non-regulated research by our institutional review board. For illustration purpose, we summarized the training data and the non-missing demographic details of participants (Table 1), and the data availability is shown in S1 Fig. We focused on predicting survival, and thus the training feature data were limited to the first 3 months of records for each participant, and predicted future survival. The 3-month training dataset was heterogeneous, e.g., participants had a variable number and occurrences of visits. Across participants, to harmonize the data, in the demographic table, we first calculated the mean of the repeated individual measurements for each feature independently. Using these means, we estimated population averages. For example, for the fvc and BMI, we calculated the mean of every measurement during first 3 months and all available time points, for each subject. Then, we used the means of each patient to calculate the overall or 3-month average and its standard deviation.

ALS Functional Rating Scale(ALSFRS) and its revision (ALSFRS-R), are validated scales of disease burden. The ALSFRS is on a 10-question scale and the ALSFRS-R on a 12-question scale. Each question ranges from 0–4. The first 9 questions are shared between the two scales. Breathing/respiratory function represents one question in the ALSFRS, but is represented by 3 questions in the ALSFRS-R. As the PRO-ACT dataset contained subjects with the ALSFRS and/or ALSFRS-R, a combined score was produced by summing up scores from related questions. Each question represents a functional task, for instance, respiratory (ALSFRS Question 10 or ALSFRS-R Questions 10–12), mouth (ALSFRS Q1-3), hand (ALSFRS Q4-5), trunk (ALSFRS Q6-7) and leg (ALSFRS Q8-9) function. Fvc1 is the first attempt to measure forced vital capacity. Fvc2 is the second attempt. Fvc: the average of forced vital capacity measurement. Fvc_percentage is the value in percentage of fvc divided by the fvc value of a non-patient, matched by gender, height and age. Age is the age of patient in years. Weight is the weight of patient in kilograms. Chloride is the concentration of Chloride in blood (mmol/L).

The PRO-ACT data was in the following format:

PatientID|datatype(form name)|feature name|feature value|feature unit|delta.

The data type included ALSFRS-R, death report (survival variable), demographics, family history of ALS, forced vital capacity, laboratory data, riluzole use, slow vital capacity, subject ALS history, treatment group, vital signs, concomitant medication use, and adverse events. The delta variable represented the time in days from the date of trial enrollment. A complete list of all 74 features and their descriptions are listed in the S2 Text. The comparison between subjects with ALSFRS-R and those without is shown in S1 Table. More information can be found at the Challenge website. For more details about the PRO-ACT dataset, please refer to PRO-ACT dataset homepage (https://nctu.partners.org/ProACT).

The data collected during the first 3 months (91.5 days) were extracted for the prediction model (Fig 1). In order to randomly divide the dataset into training and testing sets, we created individual data files for each patient ID. We randomly split the data into training and testing sets for 2-fold cross validation. To apply conventional regression modules/packages on this dataset, the data were transformed into a 2-dimensional table. The features from the dataset within the first 3 months may have been measured at different time points. Therefore, all features obtained during the first 3 months were used to calculate a 3-month mean and a slope was approximated by linear regression. Along with the first and the last records in first 3 months, we extended one numeric feature into four-element meta-features: base (baseline), end, mean, and slope. Missing values were replaced by the corresponding feature mean.

### Complete hazard ranking algorithm

The algorithm generates a single rank value for each individual, which represents the survival hazard of the individual. The values serve as prediction targets for machine learning regression models. The complete hazard ranking algorithm starts with calculating the Kaplan-Meier survival function for the dataset. The Kaplan-Meier function treats the time course of the study as a series of intervals (0t, 1t), (1t, 2t),…, (n-1t, nt) separated by every observed timestamp in the time-event dataset. The function is expressed as

$$SR(t{=}_{k}t)=\prod_{i=1}^{k}\frac{n_{i}-d_{i}}{n_{i}}=\prod_{i=1}^{k}\left(1-\frac{d_{i}}{n_{0}-\sum_{j=0}^{i-1}d_{j}-\sum_{j=0}^{i-1}c_{j}}\right)$$

where ni, di, and ci are, respectively, the number of subjects at risk, the number of occurred events, and the number of newly censored cases at the time point it.

**Fig 6:**
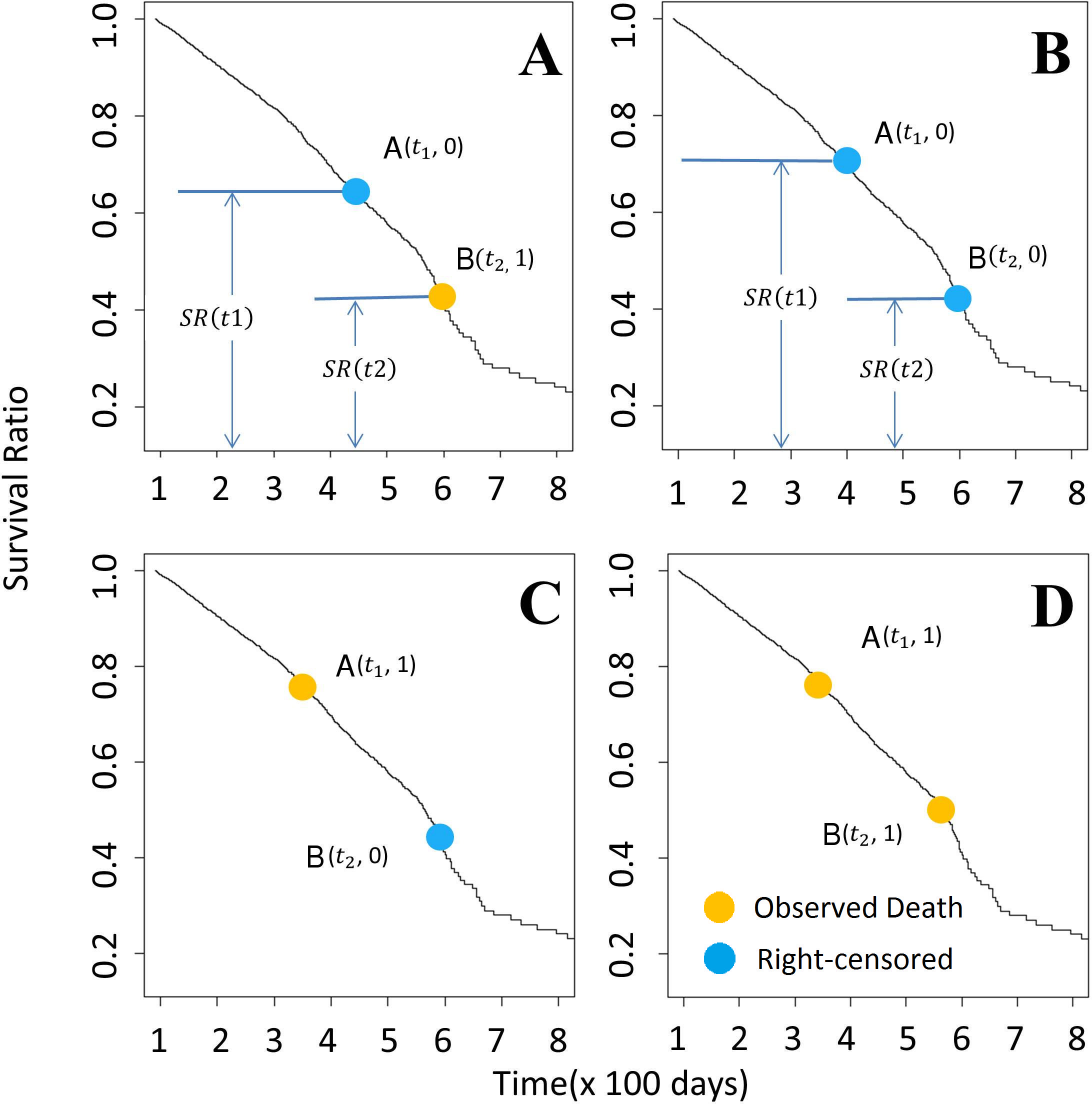
Ranking method based on the Kaplan-Meier estimator. Each panel above represents the pair-wise comparison between two participants. To compare two Patients A(t1) and B(t2), assuming t_1_ < t_2_, there are four possibilities (as shown in 6A-6D). (A) Survival time is known for patient B but is censored for patient A. The probability of patient A dying between t1 and t2 is *ρ*_*t*1,*t*2_ (*ρ*_*t*1,*t*2_ = $\frac{SR(t1)-SR(t2)}{SR(t1)}$)). In this case, we add *ρ*_*t*1,*t*2_ to rank of patient A, and (1- *ρ*_*t*1,*t*2_) to B. (B) Survival status for patients A and B are both censored. The probability of patient A dying between the time t1 and t2 is represented by *ρ*_*t*1,*t*2_. Due to the uncertainty of the both A and B, we assume that after time point t2, A and B are equally likely to survival longer than another, with the possibility of *P* = (1 − *ρ*_*t*1,*t*2_)/2. Therefore, *ρ*_*t*1,*t*2_ + (1 − *ρ*_*t*1,*t*2_)/2 and (1 − *ρ*_*t*1,*t*2_)/2 are added to the rank of A and B here, respectively. (C & D): Patient B survives longer than patient A, 1 is added to the rank of A. Yellow dots = death event of completely observed samples; Blue dots = last observation of right censored samples.

Given the Kaplan-Meier survival function, the algorithm compares all possible pairs of subjects and assigns a hazard score to each subject in the comparison. In brief, for cases where the order of events is clear for both subjects, the algorithm assigns 1 to the subject with earlier event occurrence; for cases where the order is not clear, the algorithm assigns a higher score to the subject with a higher risk of earlier event occurrence based on the Kaplan-Meier survival function. We list the detailed rules of algorithm as follows. For convenience, we denote two subjects as A and B, and their last observed follow-up time t1 and t2, respectively. The algorithm assigns scores for A and B based on the following criteria:

1. If none of the subjects are censored, the subject with a shorter time-to-event is assigned a score of 1, and the other 0 (Fig 6D). In case there is a tie, each is assigned a score of 0.5.
2. If both subjects are censored, and t1 = t2, both are assigned a score of 0.5, representing balanced uncertainty.
3. If t1 = t2, but only A is censored, A is assigned a score of 0, and B is assigned 1.
4. If t1 < t2, and an event happened to A, A is assigned a score of 1, and B is assigned 0 (Fig 6C).
5. If t1 < t2, and an event happened to B, and A is censored, A is assigned a score of ρ(t1, t2), the conditional probability that an event happened to A before the event happened to B given that no event happened to A before t1 (Fig 6A); B is assigned 1-ρ(t1, t2). The probability, ρ(t1, t2), can be calculated using Kaplan-Meier survival function:
  $$\rho (t_{1},t_{2})=1-\frac{SR(t_{2})}{SR(t_{1})}$$
6. If subjects have different observed survival time in the study, and both are censored, we rely on Kaplan-Meier estimator to calculate the score (Fig 6B). Here, we assume t1 < t2. We calculate ρ(t1, t2), the conditional probability that an event happened to A between t1 and t2 given that no event happened to A before t1, using the aforementioned equation. Then, A is assigned [1+ρ(t1, t2)]/2, and B is assigned [1-ρ(t1, t2)]/2.

Given a dataset of N subjects, each subject is compared to the other N-1 subjects. For each subject, the summation of N-1 comparison scores is assigned as the final hazard rank. By intuition, an event is more likely to happen early to a subject with a higher hazard rank than one with a lower rank. In sum, for a censored subject (E = 0) whose last observed follow-up time point is t, its hazard rank is:

$$Rank=\sum_{\forall T=t,E=0}\frac{1}{2}+\sum_{\forall T>t,E=1}\rho (T,t)+\sum_{\forall T>t,E=0}\frac{1+\rho (T,t)}{2}+\sum_{\forall T<t,E=0}\frac{1-\rho (T,t)}{2}$$

For an uncensored subject (E = 1), its hazard rank is:

$$Rank=\sum_{\forall T=t,E=1}\frac{1}{2}+\sum_{\forall T=t,E=0}1+\sum_{\forall T>t}1+\sum_{\forall T<t,E=0}\left[1-\rho (T,t)\right]$$

### Feature selection for survival prediction

For each data variable in the PRO-ACT dataset, we extracted the four-element meta-features which were then sorted according to their correlation with death-event survival. Before the calculation, we imputed all missing values with the mean of the corresponding meta-features. The top six features correlated with the ranks were selected, whereas closely related features (Pearson correlation > 0.9) were excluded (No closely related features among the selected features). Thus, if we the selected 6 features chosen based on the training set, a total number of 24 ‘elements’ of these 6 four-element ‘meta-features’ would be the maximal input for the regression models.

The Synapse PRO-ACT challenge limited the number of features that an algorithm could rely on to 6. In our model and experiments, we increased the length of the feature vector to 15 predictive features, without excluding features that may have close associations. In each round of the cross-validation tests, the correlation coefficients of all features against the prediction target (the rankings) were calculated. The most correlated feature was selected. Then, we iteratively performed the following selection steps until the maximum number of features was reached:

- Remove any features that have correlation coefficients higher than 0.9 against any selected features;
- Select the most correlated feature out of the remained ones.

Selected features were then used to build the main prediction model.

In the version of our algorithm submitted to the DREAM Challenge, we used a preset selection of features to predict the survival probability. These essential features were determined by sorting features based on their Pearson correlation with the rank, while considering their pairwise correlation, to obtain a comprehensive but non-redundant feature list. At the same time, this calculation process included the complete PRO-ACT dataset (instead of half of the dataset as we split into that training set) and the “additional training data” given by the Challenge organizers (served as a sample from the challenge evaluation data).

In order to illustrate the correlation between the first 3-month recorded features, we calculated the Pearson correlations between each pair of features (using its mean value during the first three months). Heatmap visualization and hierarchical clustering were employed (using MeV [45,46]) for exploratory data analysis and to cluster the related features into groups. As there was some incongruence in the data across subjects, the raw ALSFRS scores were used in their original scale (ALSFRS_total) or the revised scale (ALSFRS_R_total), whichever was available for the specific subject. We also summarized the baseline value for each cohort to evaluate the potential measurement bias between cohorts.

### Regression methods

To compare our method with the cox model, we applied several regression methods coupled with our ranking method as well as cox model, during the 2-fold cross validation. The normalized rank (each rank divided by the highest rank) in the training set was used as regression target variable. The first method we applied was Gaussian process regression [47]. Gaussian process regression requires a kernel function to describe the similarity of two sample points. Here we use a squared-exponential kernel function:

$$k(x,x\prime )=\sum_{i=1}^{n}e^{-\frac{{\left(x_{i}-x_{i}^{\prime }\right)}^{2}}{2l^{2}}}$$

where x and x' are two sample points of n dimensions, x_i_ is i-th feature of x, and l is the bandwidth parameter. Given the training data feature matrix X and target vector Y, the prediction target y' of the testing data point x' can be estimated with:

$$\hat{y}\prime =k\prime (K+\alpha I)Y$$

where α is the noise parameter, I is the identity matrix, and:

$$k\prime ={\left[\begin{array}{ccc} k(x\prime ,X_{1}) & \ldots & k(x\prime ,X_{n}) \end{array}\right]}^{T}$$

$$K=\left[\begin{array}{ccc} k(X_{1},X_{1}) & \ldots & k(X_{n},X_{1})\\ \vdots & \ddots & \vdots \\ k(X_{1},X_{n}) & \ldots & k(X_{n},X_{n}) \end{array}\right]$$

The algorithm was implemented in Octave. Based on the prediction result, we optimized the parameter and reached the current performance by assigning α = 5 and l^2^ = 10.

Next, we applied linear (Lasso) regression to the training dataset. The mathematical formulation of Lasso regression training is to solve:

$$\underset{\beta \in R^{p}}{min}\frac{1}{N}{\left\|y-X\beta \right\|}^{2}+\lambda {\left\|\beta \right\|}_{1}$$

where y is the training target vector, X is the training feature matrix, and β is the feature weight vector. The trained model then predicts the target value of a new sample point x' as x'β. The R package “glmnet” [48] is used, the “family” parameter is chosen to be “Gaussian”. Since the prediction would yield different results with different alpha values, we chose over-all best lambda (equals to 35) which gave the result that has the highest concordance for comparison during the cross validation.

For random forest [49], TreeBagger() function in MATLAB was adopted to generate the ensemble of bagged decision trees. The random forest model creates multiple decision trees and takes the average of their predictions. Training of each decision tree starts with sampling data points from the training set with repetition randomly and subsampling a set of features randomly. Then the model iteratively partitions the sampled data points based on the most predictive feature until a minimum number of sample points are left. Given a testing data, a decision tree finds the corresponding partition for the incoming data point and average all the training targets in the partition as the prediction. In our implementation, the rank calculated previously using our Perl script with the split training/testing datasets were read into MATLAB. During the prediction, number of trees was set to 100. The results shown in Fig 3 for random forest used a default MinLeafSize of 5 given by the function TreeBagger().

For Cox model, the time-event table was directly used as training target. Again, we used glmnet() [48] function. The parameter ‘family’ was set to ‘cox’, while lambda = 0.8 and alpha = 1.0. During the prediction, prediction (…, s = 0.6, type = “response”) was adopted.

Finally, we tested the random survival forest on this dataset. The R package “randomForestSRC” was used to perform the experiment. The model was trained using the function rfsrc() with following parameter: ntree = 1000, nodesize = 3 and family = “surv”.

### Survival time prediction methods

Three survival time prediction methods were compared in this study, one based on the Cox model, and two based on the rank-based model. Given the Kaplan-Meier curve estimated from the training dataset, the Cox model linearly rescaled the calculated hazard ratios of the training subjects to the range of [minSurv, 1], where minSurv is the lowest survival probability calculated by the Kaplan-Meier survival function. The predicted hazard ratios of the testing subjects were rescaled using the same scaling function. Because there were testing subjects predicted hazard ratios of which may be higher or lower than all training subjects, the re-scaled predicted hazard ratios might fall out of the range of [minSurv, 1] and should be clipped to the range. The clipped hazard ratios were then mapped to survival time using the inverse function of the training data Kaplan-Meier curve.

The rank-based projection method starts with constructing a Kaplan-Meier curve from only uncensored cases in the training dataset. In this case, each jump point of the curve corresponds to an observed case in the training dataset, the complete hazard rank and the survival time of which are known. All testing samples were predicted with the longest survival time that was associated with a higher hazard rank.

The rank-based interpolation method is similar to the rank-based projection method. However, instead of predicting survival time from known cases, the method constructs a linear spline function between the hazard ranks and the survival time. Such a function is guaranteed by the monotonicity between the hazard ranks and the survival time (proof omitted). All testing samples were then predicted by mapping their hazard ranks to the spline function.

### Prediction evaluation

The testing data set also went through the feature selection process as the training set. The testing features shared the same structure as the training feature dataset. The prediction of our ranking methods output the predicted rank. We used survival::survConcordance() [50] to calculate the concordance [51,52] between the predicted and the actual survival curve. The concordance is also referred to as ‘c-index’, which is given as the major performance measurement in survival analysis. Perfect prediction shows a concordance equal to 1, while 0.5 indicates a random prediction. The survival time predictions were evaluated in terms of average absolute prediction errors in days. The evaluation excluded any censored cases in the testing dataset.

### Repeated cross-validation tests

The 20x2CV tests were performed as described below:

1. Randomly shuffle the individuals and split them into two groups of approximately equal size.
2. Choose one group of individuals as the training data. Use this data to train each candidate model:
  - Add all the features to the candidate feature pool.
  - Repeat the following steps to select features from the candidate feature pool, until the maximum number of features is reached:
    ○ The feature with the highest correlation against our complete ranking is selected and used in our candidate model. The selected feature is removed from the pool. The correlation is calculated using only the training data group.
    ○ Any remained features that have got a correlation coefficient above 0.9 to the selected feature are dropped from the pool. Similarly, the correlation is calculated using only the training data group.
  - Use the selected features to train candidate model.
3. Choose the other group as the testing data. Predict the survival time (or the survival rankings) on the testing data. Evaluate the performance of all trained models. Collect the c-index score for each model.
4. Repeat Step 2 and Step 3 with training and testing data swapped. Collect the c-index score for each model.
5. Repeat Step 1–4 for 19 more times. Average all 40 c-index scores collected for each model to get the estimated performance.

## Supporting information

S1 Fig Data availability in percentage. The percentage of the available values coverage for each feature. Some related features, which share the same coverage, for instance, race information (‘race, white’; ‘race, black’; ‘race, other’; etc.) are merged into one.
(PDF)

S1 Table Summary statistics by ALSFRS groups. (PDF)

S1 Text A brief description of ALSFRS questions. (DOCX)

S2 Text Full descriptions of all features present in Pro-ACT dataset. (DOCX)
