# Supplementary material for: Complete hazard ranking to analyze right-censored data: An ALS survival study
Source: PLoS Comput Biol. 2017 Dec 18;13(12):e1005887. doi: 10.1371/journal.pcbi.1005887 (PMC5749893; doi:10.1371/journal.pcbi.1005887)
Supplement: S1 Fig — The percentage of the available values coverage for each feature. Some related features, which share the same coverage, for instance, race information (‘race, white’; ‘race, black’; ‘race, other’; etc.) are merged into one. (PDF) [file pcbi.1005887.s001.pdf]

Supporting information

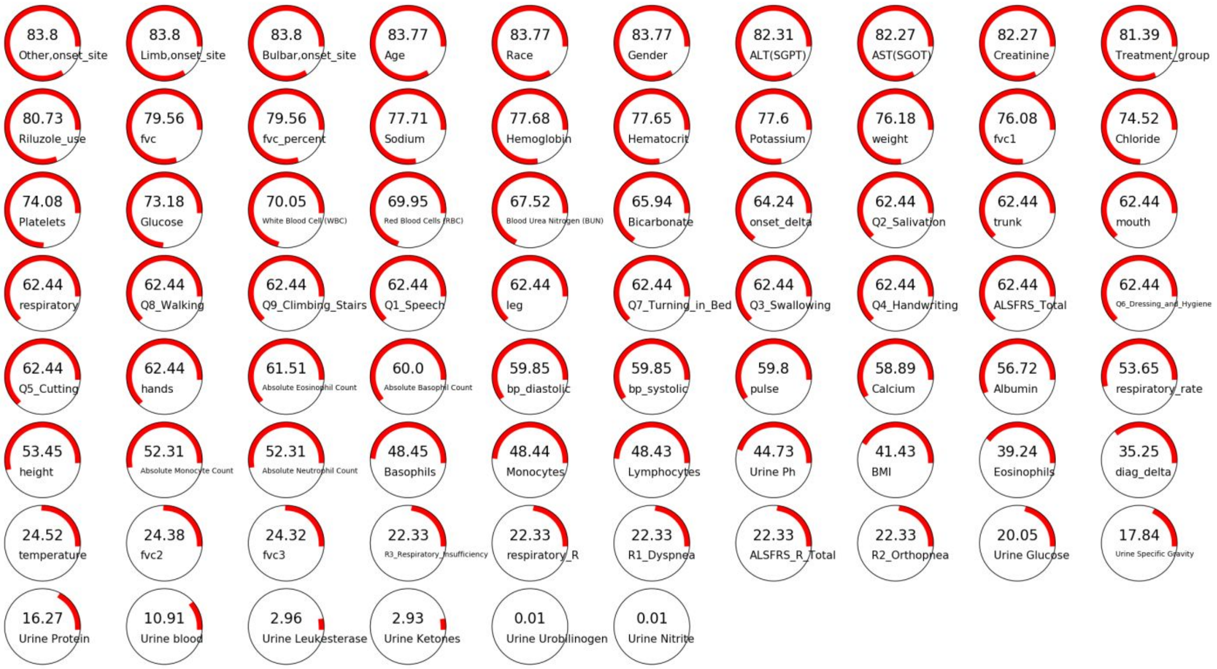

S1 Fig. Data Availability in Percentage. The percentage of the available values coverage for each feature. Some related features, which share the same coverage, for instance, race information ('race, white'; 'race, black'; 'race, other'; etc.) are merged into one.
