# Supplementary material for: Complete hazard ranking to analyze right-censored data: An ALS survival study
Source: PLoS Comput Biol. 2017 Dec 18;13(12):e1005887. doi: 10.1371/journal.pcbi.1005887 (PMC5749893; doi:10.1371/journal.pcbi.1005887)
Supplement: S1 Table — (PDF) [file pcbi.1005887.s002.pdf]

Feature	Group	ALSFRS Only Cohort (n = 3102)	ALSFRS + ALSFRS_R Cohort (n = 1736)
ALSFRS_Total	4838	29.46 ± 5.96	30.47 ± 5.11
ALSFRS_R_Total	1736	NA	38.27 ± 5.29
fvc (L)	6629	3.24 ± 1.15 (3100)	3.38 ± 1.02 (1508)
Weight (kg)	5904	73.34 ± 15.27 (3102)	76.88 ± 15.51 (1734)
fvc1(fvc trial 1 in L)	6336	3.27 ± 1.15 (3099)	3.41 ± 1.03 (1508)
mouth	4838	10.06 ± 2.37 (3102)	10.35 ± 2.18 (1736)
Q3_swallowing	4838	3.50 ± 0.73 (3102)	3.55 ± 0.71 (1736)
Q8_walking	4838	2.68 ± 0.99 (3102)	2.74 ± 0.95 (1736)
leg	4838	4.67 ± 2.31 (3102)	4.83 ± 2.23 (1736)
fvc2 (fvc trial 2 in L)	1931	2.94 ± 1.29 (1020)	3.21 ± 0.96 (909)
Chloride (mmol/L)	5830	102.49 ± 3.92 (3094)	102.82 ± 3.28 (1331)
respiratory	4838	3.68 ± 0.52 (3102)	3.67 ± 0.60(1736)

S1 Table. **Summary statistics by ALSFRS groups**
