# Supplementary material for: Complete hazard ranking to analyze right-censored data: An ALS survival study
Source: PLoS Comput Biol. 2017 Dec 18;13(12):e1005887. doi: 10.1371/journal.pcbi.1005887 (PMC5749893; doi:10.1371/journal.pcbi.1005887)
Supplement: S1 Text — (DOCX) [file pcbi.1005887.s003.docx]

**A brief description of ALS Functional Rating Scale (ALSFRS) questions**

| 1 | Speech ability |
| --- | --- |
| 2 | Salivation |
| 3 | Swallowing |
| 4 | Handwriting ability / Pen gripping |
| 5 | Cutting food with gastrostomy |
| 6 | Dressing and hygiene |
| 7 | Turning in bed |
| 8 | Walking |
| 9 | Climbing stairs |
| 10 | Dyspnea |
| 11 | Orthopnea |
| 12 | Respiratory insufficiency |
