# Supplementary material for: Complete hazard ranking to analyze right-censored data: An ALS survival study
Source: PLoS Comput Biol. 2017 Dec 18;13(12):e1005887. doi: 10.1371/journal.pcbi.1005887 (PMC5749893; doi:10.1371/journal.pcbi.1005887)
Supplement: S2 Text — (DOCX) [file pcbi.1005887.s004.docx]

**Supplementary Materials**

**Predicting ALS survival through complete ranking of right-censored data**

**Authors:** Zhengnan Huang^1^†, Stephen A. Goutman^2^†, Bhramar Mukherjee^3^, Ivo D. Dinov^4^, Yuanfang Guan^1^**, The Pooled Resource Open-Access ALS Clinical Trials Consortium

The following information are cited from DREAM ALS Stratification Prize4Life Challenge. See <https://www.synapse.org/#!Synapse:syn2873386/wiki/391434> for more detailed information.

Each subject is identified by a SubjectID and the specific assessment for this subject is identified by a record (each subject has multiple records). The features are separated into data types:

- ALSFRS(R)
- Death Report
- Demographics
- Family History of ALS
- Forced Vital Capacity
- Laboratory Data
- Riluzole use
- Slow Vital Capacity
- Subject ALS History
- Treatment Group
- Vital Signs
- Concomitant medication use
- Adverse events

**Treatment**

Treatment Group: Active or Placebo

Treatment Group delta: the time duration between the first time the patient was assessed during the trial and the time medication (or placebo) was first given. The first time the patient was assessed during the trial (typically screening visit) is indicated as Time 0. The time medication/placebo was given, is the time in days since that first day. Note that different patients started their respective trials at different days.

**Family and Medical History**

Familial: Whether or not any families had ALS (Y indicated ALS in the family, N indicates no ALS in the family)

**Demographics**

Age

Gender

Race

**Subject ALS history**

The major symptoms of ALS broadly include muscle weakness, paralysis, drooling, gagging, muscle cramps, involuntary muscle contractions/twitches called fasciculations, speech problems, and breathing problems. As ALS progresses, patients lose their ability to control voluntary muscle function. Symptoms progress from muscle weakening, twitching, and an inability to move the arms, legs, and body, into full paralysis. When the muscles in the chest area stop working, it becomes hard or impossible to breathe on one's own. ALS typically does not affect the senses (sight, smell, taste, hearing, touch).

Onset_delta: Time of symptom onset is listed in the data as [**Onset_delta**]- time in days between symptom onset and trial onset. Always negative as it happens before the trial.

Diag_delta: Time of diagnosis with ALS is listed in the data as [Daig_Delta]-time in days between diagnosis and trial onset. Always negative as it happens before the trial.

Site of disease onset: **Limb, Bulbar, Other, Limb and Bulbar, and Spine** (which is synonymous with limb onset; different terminology was used for different patients)

**Symptoms and outcome measures (FVC, SVC, ALSFRS, and Survival datatypes)**

ALSFRS_Total

ALSFRS_R_Total

Q1_Speech to Q10_Respiratory: Individual questions

FVC Normal: Forced vital capacity of a non-ALS subject

FVC1 and FVC_percent_1: First attempt of measuring FVC and its percentage from normal value

FVC2 and FVC_percent_2: Second attempt of measuring FVC and its percentage from normal value

SVC: Slow vital capacity

SVC_percent: SVC percentage from normal value

Delta_Visit_days: Time from trial onset

Status: died or not

Time_event: The days passed since trial till the status collected

**Vital signs**

Bp_diastolic: Diastolic blood pressure

Bp_systolic: Systolic blood pressure

Temperature: Body temperature

Pulse

Respiratory rate

**Concomitant Medication Use**

Medication coded: Concomitant Medications

Dose

Unit

Route

Frequency

Start delta

Stop delta

Riluzole_Use: Whether riluzole is administered

Riluzole_use_delta: Recorded time of riluzole usage

**Adverse Events**

Severity

Outcome

(Adverse events are all events recorded in the patients’ clinical records during the trial, form bruises and headaches to stroke. These events may or may not be related to the treatment at hand. They are further described by their severity [**Severity**] and their outcome [**Outcome**] of the adverse event. Information about the events is structured in the form of a hierarchical representation, where the levels in the hierarchy, listed from most specific to most generic, are named **Lowest_Level_Term**, **Preferred_Term**, **High_level_term**, and**High_Level_Group_Term**. In the representation of adverse events, we use the High_Level_Group_Term as feature name, i.e. this represents the level of granularity by which information on adverse events can be queried. Additionally, the adverse event’s start time [**Start_Date_Delta**] and end time [**End_Date_Delta**] is given.)

**Lab data**

For each lab test there is [**lab_test_name, lab_test_value, lab_test_unit, delta_days_visit**(time from the start of the trial)]. Note that a lab test result can be within the normal range and may still be relevant for predicting ALS progression (depending on where it falls within the normal range), and also that normal ranges vary according to different sources. Also note that there may be cases where mistakes were made in data entry leading to abnormally high or low (non-physiological) levels of certain measures in this database, so be mindful of this in your analysis and interpretation.

As part of our data cleaning process for the lab data, units were converted, synonymous tests appearing with different names were merged. Note the some of the rare features are only available in the training set but not in the validation set (a list is available below)

Lab test data include:

Urine:

- Urine pH- the level of acidity of the urine. Levels range between 4.5 to 8 (optimal is 6).
- Urine Protein- detects excessive protein escaping into the urine, to help evaluate and monitor kidney function, and to detect kidney damage. Normal range is 0-20 mg/dL. Note that in the database non-physiological values are most likely due to mistakes in data entry.
- Urine Specific Gravity- (sometimes listed as Specific gravity) relates to the degree of concentration of the urine, indicative of kidney function. Normal ranges are 1-1.03.
- Urine Glucose- levels of glucose in urine (measured by mg/dL). Normally, they should be zero.
- Urine WBC (white blood cells)- Should be negative. Presence may indicate higher than normal activation of the immune system (such as in the case of infection).
- Urine Leukoesterase- measuring specifically leukocyte WBC’s in the blood- should be <10 U/L.
- Urine Blood- measure of hemoglobin. Should be negative.
- Urine RBCs (red blood cells)- measure of bleeding. Should be < 3.0.
- Urine casts- another measure of bleeding. Should be negative.
- Urine Ketones- the levels of Ketone bodies found in urine, indicating starvation or carbohydrate deprivation leading to protein breakdown. Should be negative.
- Items regarding Urine Appearance include [Urine appearance, Urine Color, Urine Clarity
- Items regarding infection in Urine include [Urine Bacteria, Urine Culture and Urine Mucus].

Other measures include availability of extracts in Urine:[Urine Albumin, Urine bilirubins, Urine Hemoglobin, Urine Glucose, Urine Urobilinogen, Urine Urea, Urine Uric Acid, Urine Uric Acid Crystal, Urine Crystals, Urine Calcium Oxalate Crystals, Urine Amorphous Crystals, Urine Nitrite, Urine Potassium, Urine Sodium. (Note that some measures are listed only as – or as ‘Normal’ or on the scale of ‘Trace’, ‘Small’, ‘Moderate’ and ‘Large; without any precise numerical value).

Blood

Blood proteins:

- Albumin- a small protein produced in the liver, is the major protein in blood serum. Used to assess liver disorder or kidney disease or to evaluate nutritional status. Both increases and decreases can be significant. Normal ranges are 35- 50 g/L.
- Protein- A measure of all blood protein including Albumin. Used to assess nutritional status or to screen for certain liver and kidney disorders. Both increases and decreases can be significant. Normal ranges are 60 – 84 g/L.

Electrolytes: Measured in an electrolyte panel to assess electrolyte levels found in disease and nutritional imbalances. The concentrations of sodium and potassium are tightly regulated by the body, as is the balance among sodium, potassium, chloride, and bicarbonate. Electrolyte (and acid-base) imbalances can be present with a wide variety of acute and chronic illnesses. Both increases and decreases can be significant.

- Sodium – Abnormal levels are associated with kidney malfunction and many other pathophysiological changes. Normal ranges are 133 - 146 mmol/L (note that values are sometimes reported as lower than physiologically reasonable beyond reasonable range).
- Potassium- Normal ranges are 3.5 - 5.4 mmol/L (note that values are sometimes high beyond reasonable range).
- Bicarbonate (CO2)- Associated also with acid-base (pH) imbalance. Normal ranges are 18 - 23 mmol/L.
- Chloride- Associated also with acid-base (pH) imbalance. Normal ranges are 98 - 106 mmol/L (Note that some measures are listed only as –, without any precise numerical value).
- Anion Gap: The balance of anions and cations. Normal ranges are <11 mmol/L. If the gap is greater than normal, then high anion gap metabolic acidosis is diagnosed.
- Magnesium- measures to assess to likelihood of magnesium, poisoning. Normal ranges are or 0.6-0.82 mmol/L.

Kidney Tests: BUN and creatinine are waste products filtered out of the blood by the kidneys. Increased concentrations in the blood may indicate a temporary or chronic decrease in kidney function.

- Blood Urea Nitrogen (BUN), also known as Urea. This is a product of the kidney’s normal function found in urine. Normal ranges are 1.2-3 mmol/l. (note that values are sometimes high beyond reasonable range).
- Uric Acid- Digestive product dissolved by the kidneys. High levels might indicate kidney dysfunction or other pathophysiological changes (like gout) and are generally thought to be unhealthy within themselves. Normal ranges are 180-480 umol/L. Note that low levels of uric acid have been shown to predict worse prognosis in ALS and Uric acid is the only lab test currently known to be related to prognosis in ALS.
- Creatinine- normal ranges are 53-106 mmol/L for males. Normal BUN/ Creatinine ratios are 5-35.(note that values are sometimes high beyond reasonable range).

Liver Tests: ALP, ALT, GGT and AST are enzymes found in the liver and other tissues. Bilirubin is a waste product produced by the liver as it breaks down and recycles aged red blood cells. All can be found in elevated concentrations in the blood with liver disease or dysfunction.

- Alkaline phosphatase (ALP)- also listed as SPGT. Also associated with bone dysfunction. Normal ranges 50 - 160 U/L.(note that values are sometimes high beyond reasonable range and that some measures are listed only as –, without any precise numerical value).
- ALT (alanine amino transferase, also called SGPT)- Also associated with diseases of the biliary system. Normal ranges are 1 - 21 U/L.(note that values are sometimes high beyond reasonable range and that some measures are listed only as –, without any precise numerical value).
- Gamma-glutamyltransferase-(GGT)- Elevated serum GGT activity can be found in diseases of the liver, biliary system, and pancreas. Normal ranges are 5 - 40 U/L.
- AST (aspartate amino transferase, also called SGOT). Normal ranges are 7 - 27 U/L.(note that values are sometimes high beyond reasonable range and that some measures are listed only as –, without any precise numerical value).
- Bilirubin (Total, Direct and Indirect) - Also associated with Anemia. Normal ranges are -5-17 umol/l for Bilirubin (Total=direct+indirect), 1-5 umol/L for bilirubin (Direct) and 4 -12 for Bilirubin (Indirect).

Complete blood count: used as a broad screening test to check for such disorders as anemia, infection, and many other diseases.

- White Blood Cell (WBC)- a count of the actual number of white blood cells per volume of blood. Both increases and decreases can be significant. Normal ranges are 4.3-10.8 *109/L cells.
- White blood cell differential looks at the types of white blood cells present. There are five different types of white blood cells, each with its own function in protecting us from infection. Quantities of the various white blood cell types are listed below as either percentage or volume:
  - Neutrophils- also named Segmented Neutrophils. Normally the most abundant type of white blood cell in healthy adults, important in fighting inflammation. Normal ranges are 1.3-5.4 *109/L (Absolute Neutrophil Count) cells or 45-62% (Neutrophils).Note that some measures are listed only as – or as ‘Normal’, without any precise numerical value.
  - Band Neutrophils- are important in inflammation. Normal ranges are 0-0.7*109/L cells (Absolute Band Neutrophil count)or 3-5%(Band Neutrophils). Note that some measures are listed only as – or as ‘Normal’, without any precise numerical value.
  - Lymphocytes- make up about 25% of the total white blood cell count but can vary widely. Lymphocytes occur in two forms: B cells, which produce antibodies, and T cells, which recognize foreign substances and process them for removal. Normal ranges are 0.7-3.9 *109/L cells (Absolute Lymphocytes Count) or 16-33% (Lymphocytes). Note that some measures are listed only as – or as ‘Normal’, without any precise numerical value.
  - Monocytes - function in the ingestion of bacteria and other foreign particles. Monocytes make up 5-10% of the total white blood cell count. Normal ranges are 0.1-0.8 *109/L cells (Absolute Monocyte Count) or 3-7% (Monocytes).Note that some measures are listed only as – or as ‘Normal’, without any precise numerical value.
  - Eosinophils- are believed to function in allergic responses and in resisting some infections. Normal ranges are 0-0.5 *109/L cells (absolute Eosinophil count) or 1-3% (Eosinophils). Note that some measures are listed only as –, without any precise numerical value.
  - Basophils- normally constitute 1% or less of the total white blood cell count but may increase or decrease in certain diseases. Normal ranges are 0-0.4*109/L (Absolute Basophil Count) or 0-0.75%(Basophils). Note that some measures are listed only as – or as ‘Normal’, without any precise numerical value.
- Red Blood Cells (RBC) - a count of the actual number of red blood cells per volume of blood. Both increases and decreases can point to abnormal conditions. Normal ranges are 4.2 - 6.9 *109/L.
- RBC (red blood cell) Morphology- Abnormal morphology is found in certain blood diseases such as sickle-cell anemia.
- Hemoglobin - measures the amount of oxygen-carrying protein in the blood. Lower levels are associated with Anemia. Normal ranges are Male: 130 - 180 g/L; Female: 120 - 160 g/L.
- Hematocrit - measures the percentage of red blood cells in a given volume of whole blood. Normal ranges are Male: 45%-62%; Female: 37%-48% (out 100%).
- Mean Corpuscular Hemoglobin Count-a measure of concentration of Hemoglobin in a given volume of red blood cells (calculated by Hemoglobin/Hematocrit). Normal levels are 320-360 g/L or 32-36%.
- Mean Corpuscular Volume- Average red blood cell volume. Normal levels are 80-99 fL.
- Mean Corpuscular Hemoglobin- Average levels of hemoglobin per red blood cell. Normal levels are 27-31 pg/cell.
- Platelets- the number of platelets in a given volume of blood. Both increases and decreases can point to abnormal conditions of excess bleeding or clotting. Normal ranges are 150-350 *109/L cells (note that values are sometimes high beyond reasonable range).
- Transferrin iron-binding blood plasma glycoproteins that control the level of free iron in the blood. Both abnormally high and abnormally low levels may be indicative of Anemia. Normal ranges are 204–360 mg/dL.

Heart disease and muscle degradation

- CK (Creatine Kinase) - Increases may indicate a heart attack or other muscle damage. Normal ranges are Male: 38 - 174 u/L; Female: 96 - 140 u/L (note that values are sometimes high beyond reasonable range, and that some measures are listed only as – or as ‘Normal’, without any precise numerical value).
- Triglycerides- measured to assess the risk of developing heart disease, with increased triglyceride levels correlating with increased risk. Normal ranges depend on age: Ages 10-39 0.61-1.3 mmol/L; ages 40-59 0.77-1.7 mmol/L; age 60+ 0.9-1.7 mmol/L. Generally recommended to be kept <1.1 mmol/L (note that values are sometimes high beyond reasonable range).
- Total Cholesterol- measured to assesses the risk of developing heart disease, with increases in cholesterol correlating with increased risk. Normal ranges are 3-5 mmol/L, recommended to be kept no higher than 3.9 mmol/L.
- Lactate dehydrogenase- an enzyme involved in tissue breakdown, most commonly heart muscle damage, but also other tissues. Normal ranges are 50-150 U/L.

Blood sugar

- Glucose-level of glucose in the blood. Both increased and decreased levels can be significant. Normal ranges are 3.8-6 mmol/L (Note that some measures are listed only as Trace, Small, Moderate or Large, without any precise numeric value).
- HbA1c (Glycated Hemoglobin)- is a form of hemoglobin that is measured primarily to identify the average plasma glucose concentration over prolonged periods of time. It is formed in a non-enzymatic glycation pathway by hemoglobin's exposure to plasma glucose. Units are percentages. Level ≥ 6.5% serves as a criterion for the diagnosis of diabetes (Note that some measures are listed only as ‘Normal’, without any precise numeric value).

Mineral balance

- Calcium- routine metabolic panel to assess kidney, bone, or nerve disease. Both increased and decreased levels can be significant. Normal ranges are 2.2-2.5 mmol/L (note that values are sometimes high beyond reasonable range).
- Phosphorus –related to level of Calcium. Associated with kidney function, nutritional status, and a variety of chronic illnesses. Both increased and decreased levels can be significant. Normal ranges are 1-1.5 mmol/L.(Note that some measures are listed only as –, without any precise numerical value)

Immune response

- Hepatitis - A,B,C antigen and antibody. Positive antibody levels indicate vaccination. Positive antigen levels indicate that the person is more infectious.
- Immunoglobulins (Immunoglobulin A, G, M)-antibody isotypes. IgG is the major one found in blood. Normal range: for Immunoglobulin A 85-385mg/dL, for Immunoglobulin G 565-1765 mg/dL and for Immunoglobulin M 55-375 mg/dL.
- Gamma Globulin- a class of globulin of which Immunoglobulin G is the most common. Normal levels are 2-3 g/dL. Indicative of inflammation.
- Hormones
- TSH (Thyroid Stimulating Hormone)- A hormone that stimulates the thyroid gland to produce factors which stimulate the metabolism of almost every tissue in the body. Both increased and decreased levels can be significant. Normal ranges are 0.4-3 U/L.
- Free T3- activated by TSH. Normal ranges are 3.1-7.7 pmol/L.
- Free T4- activated by TSH. Normal ranges are 9-18 pmol/L.
- Beta HCG- a hormonal marker of pregnancy. Should be <5 U/L in non-pregnant pre-menopausal women, and <9.5 U/L in post-menopausal women.

Coagulation measures

- Prothrombin time (clotting). Normal ranges are 10-13 sec.
- International normalized ratio (INR). Normal ranges are 0.8-1.2.

Others

- Amylase- an enzyme involved in breaking down of starch. Increase levels indicate a variety of digestive problems, most commonly pancreas inflammation and ulcers. Normal ranges are 30-110 U/L.
- Salivary Amylase- Percentage of amylase that is Salivary by nature.
- Pancreatic Amylase- Percentage of amylase that is Pancreatic by nature. Pancreatic and Salivary Amylase should add up to 100%.
